# Social Identities in the Policy Process of Authoritarian Systems

**DOI:** 10.1007/s11615-022-00391-w

**Published:** 2022-04-05

**Authors:** Johanna Hornung, Ilana Schröder, Nils C. Bandelow

**Affiliations:** 1 Comparative Politics and Public Policy, TU Braunschweig, 38106 Braunschweig, Germany; 2 KPM Center for Public Management, University of Bern, 3001 Bern, Switzerland

**Keywords:** Health Policy, Environmental Policy, Russian Politics, Public Policy in Autocracies, Programmatic Action Framework (PAF), Gesundheitspolitik, Umweltpolitik, Politik in Russland, Öffentliche Politik in Autokratien, Programmatic Action Framework (PAF)

## Abstract

The integration of the social–psychological social identity approach to policy process research has recently generated new insights on policy-making. Empirical applications for established democracies and multilevel settings such as the European Union have identified five general types of social identities that are relevant for the preferences and behavior of policy actors and their stability and change over time. Social identities are based on joint memberships in social groups, such as organizations, demographic/biographical identities, sectors, locations, and informal opportunities for exchange (which may result in programmatic groups and identities). Some of these social groups, above all pluralistic interest associations and political parties, are directly related to the settings of embedded democracies. This article sheds light on the traveling capacity of the Social Identities in the Policy Process (SIPP) perspective by applying it to the Russian political system. An analysis of policy actors’ social identities in two federal ministries shows that in autocracies, interest intermediation, legitimacy, and influence on policy processes run through professional and informal groups when competing organizations and democratic institutions are absent. The results indicate that the SIPP perspective is adaptable to policy processes in different contexts but that the importance of identity types varies.

## Abstract (de)

Die Integration des sozialpsychologischen Social-Identity-Ansatzes in die Policy-Forschung hat in jüngster Zeit zu neuen Erkenntnissen über die politische Entscheidungsfindung geführt. Empirische Anwendungen in etablierten Demokratien und Mehrebenensystemen wie der Europäischen Union haben fünf allgemeine Arten sozialer Identitäten identifiziert, die für die Präferenzen und das Verhalten politischer Akteure sowie deren Stabilität und Veränderung im Laufe der Zeit relevant sind. Soziale Identitäten basieren auf gemeinsamen Zugehörigkeiten zu sozialen Gruppen, wie Organisationen, demographischen/biographischen Identitäten, Sektoren, Orten und informellen Austauschmöglichkeiten (die zu programmatischen Gruppen und Identitäten führen können). Einige dieser sozialen Gruppen, vor allem pluralistische Interessenverbände und politische Parteien, stehen in direktem Zusammenhang mit den Rahmenbedingungen eingebetteten Demokratien. Dieser Artikel beleuchtet die Reichweite der Perspektive der Social Identities in the Policy Process (SIPP), indem sie diese auf das russische politische System anwendet. Eine Analyse der sozialen Identitäten von politischen Akteuren in zwei Ministerien der Russischen Föderation zeigt, dass in Autokratien Interessenvermittlung, Legitimität und Einfluss auf politische Prozesse über professionelle und informelle Gruppen laufen, wenn konkurrierende Organisationen und demokratische Institutionen fehlen. Die Ergebnisse weisen darauf hin, dass die SIPP-Perspektive auf politische Prozesse in verschiedenen Kontexten anwendbar ist, die Bedeutung der Identitätstypen jedoch variiert.

## Introduction

The theoretical perspective of Social Identities in the Policy Process (SIPP) integrates the prominent and well-established Social Identity Theory and Self Categorization Theory into policy process research (Hornung et al. 2019). Social Identities in the Policy Process assumes that the policy process is shaped by individual actors that are guided by their subjective belonging to social groups. Actors see themselves as members of multiple social groups, which they value differently and to which they feel emotionally attached in different ways (Turner 2010; Hogg et al. 2004). How an individual thinks and acts is dependent on the social identity that is salient in a given moment.

In the past years, the SIPP perspective has been applied empirically in different contexts yet almost exclusively in settings that are characterized by democratic and pluralistic institutions and Western cultures. This poses the question of to what extent the theoretical perspective is adaptable to contexts that do not fulfil these characteristics. Taking a macro-level view, this contribution analyzes the identities of policy actors in the top levels of government in Russia to assess in what way they are characterized by overlapping identities. In doing so, the study connects to the ongoing debate in policy process research that discusses the applicability of theoretical frameworks originally developed in U.S. and European contexts in states with authoritarian structures (Heikkila et al. 2019).

The following section first gives an overview of the foundations and mechanisms of the SIPP as it is applied in policy research. Subsequently, we contrast these preconditions with the challenges posed by the current Russian political system. To examine the empirical relevance of the institutional particularities of the Russian autocracy, we look at institutions and actors of two sectors: health and environment. After describing the research design, analyzing the social group memberships of individuals in top-level positions reveals whether and which social identities of these actors overlap, revealing the relevance that SIPP unfolds in the Russian context. The article concludes with a summary and interpretation of the results and avenues for future research.

## Social Identities in Policy Research

Social Identities in the Policy Process is a theoretical perspective on public policy with an explicit social–psychological view of individuals that only recently has gained more attention in policy process research (Hornung et al. 2019). It is based on two interconnected psychological theories. The Social Identity Theory (SIT) was originally laid out by Tajfel (1974) as a theory of intergroup relations, describing and explaining behavior in favor of the group to which individuals felt they belonged in a given moment. The theory was further built upon by Tajfel’s student Turner (1982), which led to the definition of a Self-Categorization Theory (SCT) that entailed a closer focus on the individual and the way in which the individual categorization into groups steered behavior. Because both theories are based on the same foundations and assumptions, yet with different emphases, they are often referred to as the Social Identity Approach (SIA; Hornsey 2008). It is frequently applied to understand and explain social, organizational, and political changes, including political polarization and conflict (West and Iyengar 2020; Colvin 2020).

Integrating a psychological theory into policy research follows an understanding shared by many other perspectives on public policy, namely that policy processes are populated by human beings who, at least to some extent, underly the cognitive, affective, and behavioral processes of their minds. Similar to the Advocacy Coalition Framework (ACF; Jenkins-Smith et al. 2014; Henry 2011; Zafonte and Sabatier 1998) that integrated the belief systems in its rationale and the Multiple Streams Framework (MSF; Herweg, Zahariadis, and Zohlnhöfer 2017; Zahariadis 2014; Saurugger and Terpan 2016) that assumes ambiguity and time constraints as sources of bounded rationality, SIPP is an actor-centered perspective that starts from a particularly defined psychological model of the individual. Like the Programmatic Action Framework (PAF; Bandelow et al. 2021; Hassenteufel and Genieys 2021), SIPP theorizes policy actors as members of social groups that follow their salient social identity in their preferences and behaviors. These policy actors have a certain degree of influence, power, or authority in the policy process, which is a common focus of policy process theories, but this presents a first prerequisite for applying SIPP: the extent to which it is possible to identify nameable individual actors whose preferences shape the policy process.

The central idea of SIPP is that policy preferences and political action are determined by group identities. In the original psychological concepts of SIT and SCT, the understanding of group identities was open-ended because no group types were predefined. Thus, the approach presents a general theory of human behavior that is independent of political frameworks and systems. Specific to SIPP is the differentiation of five types of identities, drawing on the observation of policy processes in embedded democracies. It is yet an open question as to which of these identities are relevant and to what extent in Russian public policy, and whether and how the existing typology needs modification or supplementation when being applied to defective democracies:

Transferring this perspective of social groups and identities to the study of policy-making, it is particularly fruitful to devote attention to social groups that exert an influence on policy-specific preferences and behavior in policy processes, because this constitutes the main research interest. The five types of general identities identified by Hornung et al. (2019), four of which are observable at a macro level, fulfil these conditions. The first type of social identity is organizational identity, meaning the belonging of individuals to any type of (corporate) organization, association, or agency. In particular, the partisan identity of individuals has been proven influential for policy-related thoughts and action, e.g., when explaining voting behavior (Hornung 2022; Bornschier et al. 2021; Vogeler et al. 2020). Earlier research on partisanship and party identification profits from the understanding of political parties as social groups with internal group dynamics and intergroup relations (Clifford 2017; Huddy et al. 2015; Greene 2004).

A second type of identity that may be relevant in policy processes is that of a demographic and/or biographical identity. This type of identity is often less relevant to party politicians but is more so regarding bureaucrats and civil servants. In public administration research, Gilad and Alon-Barkat (2017) found that the conflicting identities of senior civil servants—as citizens and as professionals—influenced their preference toward policy change and a policy solution. Bureaucrats’ political attitudes and behavior are thus traceable to professional career background, biographical trajectories, and demographic characteristics (Egeberg and Stigen 2018).

Social identities that are so far less researched but potentially influential in policy processes are sectoral and local identities. The idea of sectoral identity consists of the observation that people who have worked in a specific policy sector for a longer period of time, or have dealt with a policy topic from a certain perspective, feel a belonging to the social group connected with this sectoral view. For example, a policy actor who is active in the environmental committee of the parliament or has worked in the environmental ministry might have a different view on the use of glyphosate than a policy actor with the same experience in the economics committee or ministry (Tosun et al. 2019).

The concept of local identity, on the other hand, implies that actors may feel a belonging to a group that shares the same hometown or region, or may be a representative of a local group in a given moment in time. This can be an official instance of representation, e.g., when delegates of a nation negotiate international contracts, or an implicit instance of representation, as when a transport minister funds more projects to improve the infrastructure in his region compared with other regions. National and regional identities are also subject to the study of European integration, connected to the question of what determines the formation of a European identity and what implications this has for democratic responsiveness (Westle 2003).

Besides these identities that are relatively easy to measure empirically, there is a fifth potential identity of informal groups that may shape policy processes and, not least due to its lack of transparency, be considered a threat to democratic processes of decision-making. In democratic settings, it is explicitly desired that policy actors represent different perspectives and wills of the population, whether these be organizational, demographic, sectoral, or local (Vogeler et al. 2020). If there are informal groups guiding the processes of policy-making, this presents a questionable way of presenting the democratic basis of decisions to be taken. However, informal groups often emerge from one of the other types of identity, most often that of biographical identities. In cases in which an informal group collectively forms around a policy program, which it promotes to advance the careers of its members, this is labeled a programmatic group (Bandelow et al. 2021; Hassenteufel and Genieys 2021). Programmatic groups have so far also been found in defective democracies such as Chile (Duque 2021) and Brazil (Davidian 2021).

## Social Identities—How Far Do They Travel?

At first glance, social identities are in general transferable to any institutional setting. There are no terminological references to specific cultures, political systems, or political notions. Instead, social groups exist wherever individuals come together and interact and where there are instances of intergroup behavior. Therefore, while the type and scope of social groups that are relevant in specific contexts may differ, the general hypothesis that the social identification with groups matters to the political context remains valid. Regarding the proposed types of social identities and their relevance for the policy process, however, the political context comes into play:

Building on the foundations of the SIPP perspective, this article assesses to what extent this theoretical lens is adaptable to the context of an autocracy and in what way the theoretical considerations require adjustment. To do so, it analyzes the case of Russia, which is mostly suggested to be termed an authoritarian state or autocracy (Heinemann-Grüder 2017; Lührmann et al. 2018). This contribution does not aim to connect to the question of where to align Russia in political systems’ typologies but rather to assess what this generally means for policy research. The policy style in Russia is characterized by a centralist authoritarian process of decision-making with a strong federal administration and bureaucracy (Khodachek and Timoshenko 2018). Adopted reforms and policy change often lack implementation due to regional, economic, and sectoral elites trying to boycott the reforms, which can only be and is often solved by increased centralized control (Zaytsev 2019, p. 295). The observation of a strong centralization of power is rooted in political science analyses of contemporary Russia that focus on institutions and processes of gaining, acquiring, and controlling political power, especially from an institutionalist perspective (Merkel 2018; Weßels 2007).

Formally, Russia has an electoral system, which, however, suffers from irregularities in voting and counting and from the fundamental conflict over admission of election observers, as well as from the lack of fairness. Relevant to the policy process is the observation that the party-political programs make few controversial policy-related statements. Elections and election campaigns in Russia are therefore hardly associated with policy-related disputes (Gel’man 2008), and party politics play a lesser role in social identification. While political parties present an important organizational anchor for Western democracies by providing alternatives to the electorate, and while party systems substantially structure public debate and communication, it can be expected that partisan and other organizational identities are less relevant in authoritarian systems. Nondemocratic countries do not have pluralistic structures to organize the articulation of opposing policies. Therefore, we assume organizational identities to be less important:

### H1:

Policy actors in top-level positions of autocracies are not characterized by overlapping organizational—at least not partisan—identities.

In authoritarian systems, the pathways to power and the possibility to make oneself heard is less given. Influence on decision-making relies much more on biographical connections and, potentially, money and informal networks, as bribery and a lack of transparency are often-described challenges in these settings (Ledeneva 1998; Jancsics 2019). Furthermore, the career paths of policy actors run through these channels rather than through organizational structures, such as political parties, or a merit-based system of promotion. Since personal connections are established through biographical and professional networks, we postulate the following hypothesis:

### H2:

Policy actors in top-level positions of autocracies are characterized by shared biographical identities.

While shared organizational identities should be less visible, and biographical, demographic, and informal identities more strongly characterize policy actors in top-level positions in Russia, the expectations are less straightforward for the case of sectoral and local identities. In Russia, specifically, political participation is limited and selective. There are persistent conflicts with nongovernmental organizations (NGOs; Owen and Bindman 2017), and certain business interests—especially those relevant to the Russian economy and their oligarchs—continue to exert a lobbying influence on policy-making (Rutland 2015). On the other hand, interest groups of, e.g., employees have not achieved corporatist structures (Connor 1996). For an application of the social identity perspective to the Russian policy process, this means that a trajectory in a strong policy sector and the respective businesses can be relevant to top-level policy actors. By contrast, societal actors such as NGOs and weak associations such as interest groups of employees drop out as a relevant identity.

The scope of action for regions as well as for actors working in a specific policy sector is highly dependent on whether the identity is at the same time overlapping biographically or at best informally with the state actors who occupy the top-level positions of government. While local and sectoral actors may well be influenced by their specific group belonging, the extent to which they will articulate and make heard their preferences is selective rather than systematic. Therefore, in authoritarian systems, points of contact for pluralistic interest intermediation of sectoral and local groups are strongly limited, and whether these identities find their way into politics differs from individual actor to individual actor.

### H3:

Policy actors in top-level positions of autocracies are characterized by selectively shared sectoral and local identities.

## Public Policy Research in Russia

In the public policy literature, Russia has not yet experienced as much attention as other nondemocratic regimes, such as China. At least in leading policy journals, there exist only a few empirical analyses of policy processes in Russia that apply one of the major theories of the policy process. From a macro-perspective, however, research has shown that welfare state development followed similar paths in Western democracies and Eastern states during the Cold War (Obinger and Schmitt 2011).

Policy research of post-Soviet Russia began with a very critical assessment of the health care reforms under President Yeltsin, the central contents of which were decentralization and privatization (Duffy 1997). A later survey on environmental policy has more relevance for the application of the SIPP perspective (Feldman and Blokov 2009). It reveals the perception of a close connection between the pro-Western party Yabloko and environmental issues. Research on the ACF (Weible et al. 2019) in Russia shows that public policy concentrates primarily on the energy sector and ascribes an important influence on public policy to the energy industries (Nevzorova and Kutcherov 2021). With reference to the MSF (Herweg et al. 2017), Bindman et al. (2019) outline how the expertise, credibility, and networks of major NGOs helped in setting the agenda for a family-based model of child welfare, resulting from an intentional creation of network structures between NGOs and state actors. However, the national-level restrictions on NGOs at the same time led to regionally divergent relations between subnational governments and the societal actors (Toepler et al. 2020). In line with the considerations on political participation outlined above, this suggests a weak role of policy actors’ identification with societal actors and interest advocates, and a selective importance of local identity.

These insights are, first, consistent with the expectation that biographical networks of elites (and nonelites) are strong bonds of policy actors and that organizational identities, apart from the societal sphere, are less relevant. Second, these biographical and informal identities are hypothesized to overlap with those of sectoral and local identities. Thereby, selected sectoral identities (see H3)—especially those that constitute the core of the Russian economy, such as oil and gas (Rutland 2015)—as well as specific local identities—presumably the oligarchs in Moscow and St. Petersburg—should be characteristics of central actors in the policy process. The involvement of sectoral actors in decision-making processes is thereby strongly oriented toward expertise (Heinrich and Pleines 2021), which is in line with the assumption that the executive bases its legitimacy on the expertise of individual actors in the state apparatus, alongside the collective identity (Huskey 2012).

It is therefore of particular interest to analyze the social identities that are relevant in public policymaking in Russia and to reveal the identities that bind the individual actors in top-level positions that have a direct influence on the policy process. Since identities are assumed to be a predictor of preferences and behavior, revealing the relevant identities in Russia is a first step in the research on how public policy is driven by actors from certain social groups. Existing research on identities in Russia refers to historical analyses of national identities (Davies 1997) or to class identities (Fitzpatrick 1993). In particular, what is termed an imperial identity is often referred to as an example of the totalitarian nature of the political system, in which authorities have either completely internalized and profit from the hierarchical power structure or try to use the little power they have to make personal, often monetary, profit by differently interpreting or implementing the adopted reforms (Zaytsev 2019). Especially due to this diverse importance of different social identities compared with Western democracies, Russia seems to be an ideal case for testing the traveling capacity of the SIPP perspective.

## Research Design and Methodology

To empirically assess the transferability of the SIPP perspective to the Russian political system, we conducted a comparative analysis of civil servants’ social identities in the Russian federal Ministry of Health and the Ministry of Natural Resources and Environment. The objective was to investigate whether there is evidence for particularly prevalent social identities within or across ministries that might serve as a binding element and indicate a specific importance for the appointment of civil servants in the selected policy fields in Russia or, more broadly, in autocracies. Because social identities are part of an individual’s biography, this analysis was based on biographical analyses of current civil servants in top positions in the selected ministries. This includes the ministers, deputy ministers, and, in the Ministry of Health, also advisors to the minister. To investigate whether these social identities persist in the ministries, we also analyzed the previously appointed ministers under the Putin presidency (and under Medvedev). We analyzed policy actors at the ministry level instead of the presidential administration because less biographical data are publicly available for the latter.

The Ministry of Health and the Ministry of Natural Resources and Environment were selected as cases because they are of special importance for current Russian politics. In March 2020, shortly after the World Health Organization declared COVID-19 a global pandemic, President Putin announced that the health of Russian citizens was the highest priority (Presidential Executive Office 2020). Since then, the Ministry of Health has gained political importance and taken part in deciding and coordinating the national crisis response (Reshetnikov et al. 2020), including public information, resolution and proclamation of regulations, and vaccine organization (https://covid19.rosminzdrav.ru). The Ministry of Natural Resources and Environment is of particular importance to Russia and its economy. Russia generates around 30% of its gross domestic product from the industrial sector, dominated by the fuel and energy complex (The World Bank Group 2022b). It is furthermore the second largest oil and gas producer worldwide and occupies the sixth place in coal production (BP 2021), with important interactions with security policy (Goldthau et al. 2020). Russia’s fuel export counted for 50% of total merchandise exports before the pandemic (The World Bank Group 2022a), making the country economically vulnerable to international agreements and oil prices (Filimonova et al. 2020).

To identify civil servants’ social identities, we analyzed digitally accessible data on their biographies. We interpreted these biographical data and intersections between individuals as potential identification with related social groups. This methodological approach has limitations that arise from the lack of transparency of authoritarian political systems. Compared with embedded democracies, knowledge about the government’s discussions, networks, and power distributions is low. Policy processes are less transparent, preferences are less publicly visible, and identification of political positions via media is limited by the government’s control over newspapers and television (Uldanov et al. 2021). Furthermore, political actors are less accessible for interviews than in political systems with transparency rules. Even though recent research indicates that policy narratives and proposals can be analyzed in Russia at a local level (Schlaufer et al. 2021a, b), the challenges of collecting data on preferences and behavior of policy actors in higher positions in authoritarian systems remain.

These conditions specifically impede the application of SIPP, which requires the traceability of policy processes, associated actors, and their social identities. Since we can hardly understand the development of a specific policy and the associated policy makers in Russia on the basis of publicly available information, we decided to broaden the theoretical focus of the SIPP on the policy process and focus on actors in high political positions and their social identities. According to the social–psychological theoretical foundations of the SIPP, social identities shape the behavior of individuals only if they are salient. Since capturing the salience of social identities relies on internal psychological processes that are difficult to measure, our analysis focuses on commonalities and differences of potentially salient social identities and the extent to which they may be characteristic of certain ministries or authoritarian political systems. By gathering information on social groups and related identities of ministerial bureaucrats, we can identify a binding element of policy actors in top-level positions who are involved in political decision-making.

The biographical data of the civil servants were obtained from the websites of the ministries (MNR 2021; MINZDRAV 2021) and journalistic portals and publishers such as Zdrav.Expert (2021), Kommersant (2021), TAdviser (2021), Tass (2021), and the “Who is Who?” section of Delovoj Peterburg (2021). The analysis examines the four formal identities of the SIPP approach. This includes demographic identity, local identity, organizational identity, and sectoral identity.

For demographic/biographical identity, we assessed the sex, age, and professions of the civil servants. The local identity was derived from information on the regions the actors either lived, studied, or worked in for a long period of time or at several points in time. If there was relevant information on more than one region, we assigned multiple (potential) local identities. Individuals who either changed their place of residence frequently or for whom no biographical data were available were not assigned a local identity. The organizational identity was based on a civil servant’s membership in organizations or on affiliation with them. This includes, inter alia, (non)governmental institutions and political parties. We took into account only organizations and related identities that were relevant for the analysis, i.e., those with connections to the policy sector of the respective ministry, to the Russian government, to other social identities of the respective individual, or to other civil servants. The sectoral identity was partly linked to the organizational identity of the studied individuals. One may argue that all people being part of, e.g., the Ministry of Natural Resources and Environment have a sectoral identity related to the ministry. However, there may be civil servants who only recently joined the ministry and/or used to work or engage in different sectors previously. To decide on the potentially most relevant sectoral identity, we therefore combined information on the civil servants’ education and professions as well as on the period they spent studying/working in fields related to a certain sector. The assignment of multiple sectoral identities was therefore possible if someone, for instance, studied medicine and law, worked in the legal sector for one decade, and then worked as a physician for several years. If actors shared multiple formal identities, i.e., biographical interlinkages, we interpreted these overlaps as indicators for informal networks (informal identity) between these civil servants.

## Empirical Results: Social Identities of Civil Servants in the Russian Federal Ministry of Health and the Ministry of Natural Resources and Environment

The empirical investigation of civil servants’ biographies partly faced the challenge of insufficient publicly available data, particularly among advisors to the minister of health. Nevertheless, enough data were available for most civil servants under study. In total, 18 individuals from the Ministry of Health and 11 individuals from the Ministry of Natural Resources and Environment were included in the analysis. The biographical data and related social identities are presented in Tables 1 and 2, respectively. We assume that the importance of social identities in the policy process can be confirmed when there is evidence for civil servants having several overlapping social identities with other individuals working in the same ministry. The more social identities seem to overlap in a personnel team, the more likely these specific identities are constitutive of the ministry and policy sector of investigation. If the individuals working in the ministries do not share social identities specific to the ministry, it can be assumed that other factors are driving the (collaborative) work in that organization.

**Table 1:** Social identities in the Ministry of Health

Position in the ministry	Name	Organizational identity	Professional identity	Sectoral identity	Local identity
Advisor to the minister	Bychkov, S. V.	RF Ministry of Foreign Affairs RF Ministry of Health	Medic	Health	–
State secretary and deputy minister	Dronova, A. V.	RF Ministry of Justice (> 10 years) RF Ministry of Health	Lawyer	Legal Sector	Moscow Oblast
First deputy minister	Fisenko, V. S.	Roszdravnadzor Ministry of Health in Primorsky Krai RF Ministry of Health	Pharmacist	Health	Primorsky Kraj
Advisor to the minister	Fleck, V. O.	RF Accounts Chamber RF Ministry of Health (> 15 years)	Public administrator Medic	Health	Moscow Oblast
Deputy minister	Glagolev, S. V.	Roszdravnadzor (> 15 years) RF Ministry of Health	Medic	Health	Moscow Oblast
Former minister (2007–2012)	Golikova, T. A.	RF Ministry of Finance (> 15 years) RF Ministry of Health, FFOMS RF Accounts Chamber Party: United Russia	Economist	Civil service	Moscow Oblast St. Petersburg
Deputy minister	Gridnev, O. V.	RF Ministry of Health Presidential Academy	Economist Medic	Health	Moscow Oblast
Deputy minister	Kamkin, J. G.	RF Ministry of Health	Medic	Health	Moscow Oblast
Deputy minister	Khorova, N. A.	RF Ministry of Health (> 15 years)	Economist	Civil service	Samara Oblast Moscow Oblast
Current minister (since 2020)	Muraschko, M. A.	Roszdravnadzor Ministry of Health in Komi Republic RF Ministry of Health	Medic	Health	Komi Republic
Advisor to the minister	Panin, A. I.	Roszdravnadzor RF Ministry of Health	–	Health	–
Deputy minister	Pugachev, P. S.	Rosstandart RF Ministry of Communications RF Ministry of Health	–	Technology	Moscow Oblast
Advisor to the minister	Ryzhov, D. L.	RF Ministry of Health	Military colonel	Army	–
Deputy minister	Salagaj, O. O.	RF Ministry of Health (> 10 years)	Lawyer Medic	Health	Irkutsk Oblast
Former minister (1999–2004)	Schevtschenko, J. L.	RF Ministry of Health (> 15 years)	Medic	Health	St. Petersburg Moscow Oblast
Deputy minister	Semyonova, T. V.	RF Ministry of Health (> 10 years)	Medic	Health	Moscow Oblast
Former minister (2012–2020)	Skvortsova, V. I.	National Stroke Association (NABI) RF Ministry of Health (> 10 years) Presidential Academy	Economist Medic	Health	Moscow Oblast
Former minister (2004–2007)	Surabov, M. J.	Konversbank JSC MAKS Medical Insurance Company RF Ministry of Health	Economist	Civil service Finance	–

**Table 2:** Social identities in the Ministry of Natural Resources and Environment

Position in the ministry	Name	Organizational identity	Professional identity	Sectoral identity	Local identity
Deputy minister	Anoprienko, S. M.	RF Ministry of Natural Resources and Environment, RF Ministry of Emergency Situations, Federal Property Management Agency, Presidential Academy	Public administrator Economist	Civil service	Moscow Oblast
Former minister (2001–2004)	Artjuchov, W. G.	RF Ministry of Natural Resources and Environment Party: United Russia	Economist Engineer	Finance Transport	–
Former minister (2012–2018)	Donskoj, S. J.	RF Ministry of Energy, RF Ministry of Natural Resources and Environment (> 10 years)	Engineer	Oil & gas	Moscow Oblast
Deputy minister	Kerimov, M. K.	RF Ministry of Natural Resources and Environment	Lawyer	Environment	–
Former minister (2018–2020)	Kobylkin, D. N.	RF Ministry of Natural Resources and Environment Party: United Russia	Public administrator Engineer	Oil & gas	Ural Oblast
Current minister (since 2020)	Kozlov, A. A.	Ministry of Housing and Communal Services of the Amur Oblast, RF Ministry for the Development of the Russian Far East and Arctic, RF Ministry of Natural Resources and Environment Party: United Russia	Lawyer	Coal	Amur Oblast
State secretary and deputy minister	Radchenko, S. Y.	RF Ministry of Natural Resources and Environment (> 20 years)	Economist Lawyer	Environment	Moscow Oblast
Deputy minister	Tetenkin, D. D.	Ministry of Housing and Communal Services of the Amur Oblast, RF Ministry for the Development of the Russian Far East and Arctic, RF Ministry of Natural Resources and Environment	Engineer	Coal	Amur Oblast
Former minister (2004–2012)	Trutnev, J. P.	RF Ministry of Natural Resources and Environment Party: United Russia	Public administrator Engineer	Oil & gas	Perm Krai
First deputy minister	Tsyganov, K. A.	RF Ministry of Natural Resources and Environment, RF Ministry for the Development of the Russian Far East and Arctic, Presidential Academy	Economist Lawyer	Civil service	Moscow Oblast
Deputy minister	Yastrebov, S. N.	RF Ministry of Natural Resources and Environment	Engineer	Civil service	Yaroslavl Oblast

The empirical analysis of civil servants’ identities shows that identity types found in embedded democracies are also present in Russia. However, some identity types seem to be more important for autocratic than for democratic systems. Particularly striking is the relevance of ministry-specific professional identities, which—in contrast to previous SIPP research—should be differentiated from demographic identity. While the SIPP perspective adds to an understanding of the personnel in both Russian ministries, the Ministry of Natural Resources and Environment, possibly because it is more relevant for the presidential administration regarding its importance for the country’s economy, includes more civil servants who cannot be explained by ministry-specific identities but by a closer connection to the president, for instance through their education, party affiliation, or the personnel pool of the president. The results are discussed in detail in the following.

### Organizational Identity

The affiliation with different political parties and associated identification with different (political) values, programs, and goals is a characteristic that particularly distinguishes policy actors in embedded democracies. In the two Russian ministries under study, the party affiliation of the actors, if existent at all, is limited to “United Russia,” the party supporting the president. Out of 18 actors in the Ministry of Health, only former minister Golikova, who was appointed vice prime minister afterward, is a member of the ruling party. In the Ministry of Natural Resources and Environment, four of 11 actors are party members of United Russia, including three of four analyzed ministers, suggesting a stronger connection to politics in this ministry.

Although party affiliation plays a lesser role in health policy, the common identification of the members of the health ministry is characterized by a specific health policy organization, the Federal Service for Surveillance in health care (Roszdravnadzor), which is responsible for controlling and supervising the health care system. Two of the deputies, one advisor, and the current health minister held positions at the Roszdravnadzor prior to their work at the Ministry of Health, all with timely overlaps. In the Ministry of Natural Resources and Environment, no organization could be identified that would suggest an intraministerial identification similar to Roszdravnadzor in Russian health policy.

In both studied ministries, the studied civil servants had previously often held positions in other ministries. Working for the state thus supports further work for the state, even if the policy sector changes. Proximity to the state apparatus is also evident through training at the Russian Presidential Academy of National Economy and Public Administration (RANEPA), which applies to two actors in each of the two ministries. However, in the Ministry of Health, the training at the presidential academy is rather an additional qualification to medical training. In the Ministry of Natural Resources and Environment, on the other hand, actors who graduated from the presidential academy have no other organizational or professional connection to the respective policy field and work purely as civil servants. Another connection of the Ministry of Natural Resources and Environment to state politics is shown by the former minister Kobylkin, who is now head of the State Duma Committee on Ecology, Natural Resources, and Environmental Protection. Such connections to the State Duma are not found in the Ministry of Health, suggesting that thematic expertise is more important in this ministry than civil service and proximity to state politics.

### Demographic and Professional Identities

In the original version of SIPP, demographic (sex, age) and biographical (education, profession) identities were subsumed under a common heading. However, the analysis of Russian policy actors suggests that it is analytically fruitful to separate these types of identities, as they are of different importance to the policy process. This is mainly because professions and, with them, biographical trajectories can be actively chosen and are supposed to have a stronger effect on socialization than demographic characteristics.

Regarding demography, the political actors investigated in the Ministry of Health ranged in age from 38 to 65. In the Ministry of Natural Resources and Environment, the age range is between 30 and 67. This distribution does not suggest that age, as part of demographic identity, is a unifying criterion for the staff of either ministry. If we look at sex, there is a predominance of men in both ministries. Ten of the 11 studied policy actors in the Ministry of Natural Resources and Environment are men. In the Ministry of Health, of the 20 current and former ministers and current deputies and advisors, 14 are men.^1^ Although this observation shows that high positions in these two Russian ministries tend to be occupied by men, this does not necessarily indicate the influence of a social identity specific to these ministries, as this can as well be a criterion for political positions in Russia in general. If we take Russia’s presidential administration as an example for comparison, a ratio of two women to 32 men confirms the sex distribution of the investigated ministries. The Ministry of Culture is the only one out of 21 ministries headed by a female minister (Ministry of Culture of the Russian Federation 2021). The generally unequal distribution of political positions between men and women in Russia therefore does not indicate a ministry-specific demographic identity in the health or resource ministry. Furthermore, we cannot conclude that this is a characteristic specific to authoritarian systems, as women are also less represented in Western democracies (Thomsen and King 2020; Fernández and Valiente 2021).

Apart from these demographic intersections, our analysis of Russian policy makers’ biographies and social identities indicates that education and profession (originally also part of the demographic identity) are highly relevant for understanding the two ministries under study and, possibly, autocratic systems in general. Empirically we assessed the biographical information on education/profession in the same way as the other data, originally to include it in the demographic identity. However, even though sex as an indicator for demographic identity is dominant in the analysis of Russian civil servants, it is neither a ministry-specific nor an autocracy-specific feature for the appointment of policy actors. In contrast, we found that ministry-specific formal education and professional experiences are shared by the biggest proportion of the studied individuals in the respective ministry. Therefore, we analytically separated shared professional identity from other less influential demographic criteria and understand it as a separate type of social identity.

The professional identity comprises a professional and/or scientific education specific to the ministry and policy sector in which the civil servant works. These professional identities are evident in both ministries. The dominant professional identity specific to the Ministry of Natural Resources and Environment is engineering, studied by six of 11 actors. The current minister, Kozlov, is the only one of five ministers since 2001 who is not an engineer. More than half of the investigated civil servants in the Ministry of Natural Resources and Environment have a background in multiple disciplines, including economics, law, and public administration. While economists (five out of 18), lawyers (two out of 18), and one public administrator are also found in the Ministry of Health, most civil servants in this ministry (11 out of 18) have at least one degree in medicine or pharmacy. Of the five ministers since 1999, two were physicians, two were economists, and one had degrees in both professions.

This finding is in line with existing studies that understand technocratic governing structures as a—yet unsuccessful—strategy to outweigh flawed input legitimacy by an increased output legitimacy (Huskey 2012; Jones 2019). Governments then rely on the expertise of civil servants who have specialized training in the policy sector they are concerned with, and this professional identity has a substantial influence on public policy. Additionally, in less pluralistic countries, these identities can ensure that sectoral interests are mediated through these individuals, as other points of contact do not exist for interest associations.

### Sectoral Identity

The examined policy actors have common issue-nonspecific sectoral identities and distinct ministry-specific identities. Both ministries employ general civil servants who are not part of a specific policy sector but are characterized by having worked for the government for at least one decade. These individuals share intersections such as graduating from the presidential academy, being appointed assistant to the president, or being included in his personnel pool. Two of the five previous health ministers previously held senior positions in Russia’s federal system, such as mayors, governors, or ministers in federal subjects. The same applies to two of three of the previous ministers of the Ministry of Natural Resources and Environment. This suggests that holding prior positions in government services increases the chance of career advancement into federal ministries across policy sectors.

While this sectoral identity is shared only by a small proportion of the investigated actors, ministry-specific sectoral identities dominate in both ministries. The Ministry of Natural Resources and Environment is mainly staffed by actors who belong to the oil and gas or the coal sector. Although the strong representation of these energy sectors is rather nontypical for an environment ministry from the perspective of Western democracies, this shows that the prioritization of environmental issues in Russia, as the name of the ministry suggests, focuses more on natural resources than, for example, on climate protection. In the Ministry of Health, almost 70% of the analyzed actors have been involved in the health sector in various aspects of their lives, often over decades, for instance, through their family background, education, or professional career. This ministry is thus dominated by people who have both theoretical and practical experience in medicine and can contribute ministry-specific expertise.

There are civil servants who do not fit the sectoral identity pattern of their colleagues or ministers. These actors have served in the Secretariat of the Supreme Soviet of the USSR (Artjuchov), were part of the presidential administration (Pugachev), have a high military rank in the armed forces of the federation (Ryzhov), or have pursued a career in the federal Ministry of Justice (Dronova). All of these positions are characterized by proximity to state politics, which suggests that the policy actors were assigned their ministry positions not because of their expertise in the respective policy field but because of other state services or personal relationships. In this context, informal identity could serve as an explanatory factor.

### Local Identity

Since the investigated federal ministries are in Moscow, all civil servants working there share a potential local Moscow identity. To refrain from this nondifferentiated view, we appointed Moscow as the local identity only if the civil servant had either spent several years in institutions located in Moscow or had been there over multiple stages in their life. For example, deputy Anoprienko in the Ministry of Natural Resources and Environment was assigned a Moscow identity because he completed his university education in the capital and had worked in other federal agencies and ministries in Moscow before joining his current ministry. As a counterexample, the current minister of the same ministry, Kozlov, despite now working in Moscow, was assigned a local identity for the Amur Oblast, having lived and worked there for almost 20 years, including holding the positions of mayor and governor of the oblast. Comparing the local identities of the actors in the Ministry of Natural Resources and Environment with those in the Ministry of Health, the Moscow identity in the latter can be identified for almost every second person even before their activity in the Ministry. However, this can be explained by a long-term affiliation to any federal ministry, as Moscow is a centrum for political power and federal administration instead of a specific of the health ministry. In the Ministry of Natural Resources and Environment, several civil servants come from regions that are rich in natural resources, for instance Amur, Ural, and Perm. This is mirrored in the sectoral identity of the respective actors.

### Informal Identity

As illustrated above, some studied bureaucrats fall outside the pattern of shared identities that shape the respective ministry. These individuals served in other presidential services prior to working in the ministry, leading to the assumption of shared informal identity. We assume that in these cases, informal relationships are more important to the civil servant’s position than organizational or professional identity and associated expertise. Despite the general difficulty of assessing informal identities and relationships with publicly available data, it was possible to identify connections between two actors in each ministry that suggest a shared informal identity that influences their positions in the ministry.

The Minister of Natural Resources and Environment, Kozlov, who has been in office since the end of 2020, and his deputy, Tetenkin, have had this working relationship (director and deputy director) for over 10 years. Their cooperation started in the coal company “Амурcкий уголь” (Amur’s coal) in the city of Rajchihinsk, Amur oblast, where Tetenkin was deputy of the general director Kozlov from 2006. When Kozlov was later appointed Minister of Housing and Communal Services of Amur Oblast in 2011, he appointed Tetenkin as his deputy in 2012. In this way, Tetenkin, a civil engineer, moved from the coal sector to a ministerial office. When Kozlov was mayor of a city in the Amur Oblast from 2014 to 2015, Tetenkin also switched positions and was Kozlov’s deputy. From 2015 to 2018, Kozlov was governor of Amur. He again made his longtime companion his deputy and gave him a ministerial post. In 2018, Kozlov was appointed Minister of the Russian Federation for the Development of the Far East and the Arctic and took Tetenkin with him to Moscow to serve in the position of his advisor. Since 2021, they have continued their working relationship at the Ministry of Natural Resources and Environment. Kozlov and Tetenkin have thus shared an informal connection for over 15 years that goes beyond, for instance, the coincidental affiliation to the same organization. Their (working) relationship transcended organizations, sectors, and locations.

A similar relationship can be found in the Ministry of Health between the current health minister, Murashko, and his advisor, Panin. Before being appointed health minister of the Russian federation, Murashko was head of Roszdravnadzor from 2013 to 2020, His current advisor, Panin, was already his advisor at Roszdravnadzor at this time. Murashko and Panin were appointed health minister and advisor, respectively, in the same year. This suggests a shared personal, informal identity that started long before their work at the ministry.

## Conclusion

This contribution has outlined the applicability of the SIPP perspective to the Russian autocracy. By analyzing the social identities that policy actors in top positions of the Russian political system share, we have shed light on the types of identities that are potentially more relevant in autocracies than in democracies, and vice versa. In detail, the empirical results show that the organizational partisan identity is not a central characteristic of the individuals occupying these positions (confirming H1) but that it is rather the sectoral and professional identities that are present and shared by them (confirming H2, partially confirming H3). From this finding follows the recommendation to split the type of professional identity from the original category of demographic identity. In authoritarian settings, the technocratic idea of public policy-making leads to a substantial relevance of professional and sectoral trajectories as sources of expertise and as social identities, but this is different from a demographic identity of actors. The relevance of a ministry-specific local identity could not be confirmed for the Ministry of Health. Some civil servants in the environmental ministry are related to regions rich in natural resources, which may support a high position in the ministry when it is accompanied by a ministry-specific sectoral identity.

In terms of the theoretical fit, there is nothing to argue against the assumptions that individual actors also substantially shape the policy process in Russia and that individual policy actors are bound by social identities. What differs, however, and needs a certain degree of adaptation is which types of social identities are relevant. In autocracies, the political institutions determine the potential social groups that are relevant. The application to the authoritarian setting of Russian public policy performed in this contribution has revealed that in contrast to democracies, organizational identities play a lesser role and that it is instead professional and sectoral identities that shape public policy. However, the relevance of education as it is present in French programmatic action studies through the National School of Administration (École Nationale d’Administration) (Hassenteufel et al. 2020) cannot be confirmed for Russia, despite the existence of a comparable presidential academy.

How identities are operationalized and measured poses a substantial challenge to the traveling capacity of the social identity perspective to autocracies. Thereby, SIPP does not pose ontological challenges to the application in autocracies, but epistemological and methodological challenges. Our analysis is limited to publicly available data for individual policy actors at the formal top level of government. The access to comprehensive data on policy processes clearly presents a challenge to public policy research in authoritarian settings, to which future research hopefully may find answers. This contribution at least presents one way of effective data collection and is able to give meaning to these observations.

A further limitation lies in the fact that the contribution does not look at policy outputs or establish a link between adopted policies and identities. The core interest was an identity-based analysis of individual policy actors to draw conclusions on the relevance of different memberships in social groups in Russian public policy. This opens avenues for further research on the question of how social identities link to public policies, how policy programs are developed under different degrees of democratic and authoritarian settings, and how—depending on the setting—the existence of different social group identifications is tolerated or enforced, or how conflicts between them arise.

Nevertheless, researching social identities in the policy processes of autocracies presents an added value to the study of public policymaking: One of the core interests of research on social identities lies in conflicting group identities, not only regarding polarization but also policy change. This is supposed to result from actors changing their preferences without having changed their standpoint through policy learning, economic interests, or obviously rational cost–benefit considerations. When actors with conflicting identities decide to represent the preferences of one group instead of another that they are a member of, an essential explanatory factor is which one they choose, why, and when. This type of change is even more exciting to observe in authoritarian systems because conflicting identities are not initially considered. The view from the outside suggests that an identity is hierarchically set by the center of power in the authoritarian state, and so are preferences and behavior; SIPP makes it possible to confirm this assumption and also to explain policy change by looking at competing identities.

Beyond policy research, the results can also provide impetus for democracy research. With Russia’s autocracy, the case selection has made it possible to highlight the different types of identity that prevail in authoritarian political systems. In Russia, specific identity types could be identified that differed across ministries and thereby across policy sectors. Analogous to embedded democracies, some of these were rather informal. It will be interesting not only to examine these findings in other autocracies but to view the differing identities as a systematic distinction between democracies and autocracies. It can be assumed that in some autocracies, organizational identities (for example, through membership in the dominant party, the secret service, or the army) can also play a role. The identity concept thus offers an interdisciplinary view on the comparison of political systems that goes beyond a mere comparison of institutions and can equally stimulate the differentiation of political systems as well as their evaluation in terms of legitimacy and policy processes.

## Footnotes

1 One of the 20 actors, Oksana Igoryevna Guseva, is not included in the biographical analysis because there was not sufficient digitally accessible biographical data on her.
